# Energy metabolism and muscle activation heterogeneity explain ${\dot{V}}_{O_{2}}$ slow component and muscle fatigue of cycling at different intensities

**DOI:** 10.1113/EP090444

**Published:** 2023-01-17

**Authors:** Paulo Cesar do Nascimento Salvador, Eduardo Marcel Fernandes Nascimento, Diego Antunes, Luiz Guilherme Antonacci Guglielmo, Benedito Sérgio Denadai

**Affiliations:** ^1^ Physical effort Laboratory Sports Centre Federal University of Santa Catarina Florianopolis Brazil; ^2^ Leonardo da Vinci University – Uniasselvi/VITRU Education Indaial Brazil; ^3^ Human Performance Laboratory São Paulo State University Rio Claro Brazil

**Keywords:** efficiency, muscle fatigue, oxidative metabolism, oxygen extraction, oxygen uptake slow component

## Abstract

This study aimed to explain the ${\dot{V}}_{O_{2}}$ slow component (${\dot{V}}_{O_{2}\mathrm{sc}}$) and muscle fatigue during cycling at different intensities. The muscle fatigue of 16 participants was determined through maximal isokinetic effort lasting 3 s during constant work rate bouts of moderate (MOD), heavy (HVY) and very heavy intensity (VHI) exercise. Breath‐by‐breath ${\dot{V}}_{O_{2}}$, near‐infrared spectroscopy signals and EMG activity were analysed (thigh muscles). ${\dot{V}}_{O_{2}\mathrm{sc}}$ was higher during VHI exercise (∼70% vs. ∼28% of ${\dot{V}}_{O_{2}}$ reserve in HVY). The deoxygenated haemoglobin final value during VHI exercise was higher than during HVY and MOD exercise (∼90% of HHb physiological normalization, vs. ∼82% HVY and ∼45% MOD). The muscle fatigue was greater after VHI exercise (∼22% vs. HVY ∼5%). There was no muscle fatigue after MOD exercise. The greatest magnitude of muscle fatigue occurred within 2 min (VHI ∼17%; HVY ∼9%), after which it stabilized. No significant relationship between ${\dot{V}}_{O_{2}\mathrm{sc}}$ and muscle force production was observed. The τ of muscle ${\dot{V}}_{O_{2}}$ was significantly related (*R* ^2^ = 0.47) with torque decrease for VHI. Type I and II muscle fibre recruitment mainly in the rectus femoris moderately explained the muscle fatigue (*R* ^2^ = 0.30 and 0.31, respectively) and the ${\dot{V}}_{O_{2}\mathrm{sc}}$ (*R* ^2^ = 0.39 and 0.27, respectively). The ${\dot{V}}_{O_{2}\mathrm{sc}}$ is also partially explained by blood lactate accumulation (*R* ^2^ = 0.42). In conclusion muscle fatigue and O_2_ cost seem to share the same physiological cause linked with a decrease in the muscle ${\dot{V}}_{O_{2}}$ and a change in lactate accumulation. Muscle fatigue and ${\dot{V}}_{O_{2}\mathrm{sc}}$ are associated with muscle activation heterogeneity and metabolism of different muscles activated during cycling.

## Abstract (highlights)

- **What is the central question of this study?**
  What are the physiological mechanisms underlying muscle fatigue and the increase in the O_2_ cost per unit of work during high‐intensity exercise?
- **What is the main finding and its importance?**
  Muscle fatigue happens before, and does not explain, the ${\dot{V}}_{O_{2}}$ slow component (${\dot{V}}_{O_{2}\mathrm{sc}}$), but they share the same origin. Muscle activation heterogeneity is associated with muscle fatigue and ${\dot{V}}_{O_{2}\mathrm{sc}}$. Knowing this may improve training prescriptions for healthy people leading to improved public health outcomes.

## 1 INTRODUCTION

Reductions in work capacity during cycling at different intensities can be measured as a decrease in force or power production after a specific task or an increase in energy requirement to maintain the same force or power production during exercise (Grassi et al., 2015). Classically, muscle fatigue can be analysed as the loss of capacity to produce maximal voluntary force in an isokinetic dynamometer, or alternatively, the loss of power output or torque in a cycle ergometer equipped with an instantaneous switching from constant work rate to isokinetic (constant pedal cadence) cycling (Coelho et al., 2015; Enoka & Duchateau, 2008).

Central fatigue corresponds to a failure (or compromised willingness) of the central nervous system to activate the working muscles (Blain & Hureau, 2017). Peripheral fatigue can be described as a fall in the contractile power of the muscle without an impairment of conduction from nerve to muscle due to biochemical factors in the myocytes (Merton, 1954). Thus, the extent to which muscle fatigue is documented during exercise depends upon the methodology of neuromuscular assessment (Froyd et al., 2013). It is possible to argue that maximal voluntary contraction (MVC) is preserved during constant work rate cycling exercise with reductions being stable during the main part of the task (Gajanand et al., 2020; Keir et al., 2016). It seems that the major changes in muscle function occur within the first 40% of the exercise (Froyd et al., 2013). For instance, peripheral fatigue develops early during constant‐load intense cycling and is compensated by additional motor drive, while central fatigue appears to be associated with task failure (Decorte et al., 2012). Thomas et al. (2016) have reported that the magnitude of peripheral and central fatigue after cycling is dependent on exercise intensity and duration, respectively. These authors found similar reductions in MVC in different high‐intensity cycling trials, although peripheral fatigue showed greater reductions after higher exercise intensities.

Over the past decades, numerous studies have aimed to understand the physiological mechanisms underlying a loss of work efficiency or an increase in the O_2_ cost per unit work during constant‐load exercise above the gas exchange threshold (GET) (Borrani et al., 2001; do Nascimento Salvador et al., 2018; Gajanand et al., 2020; Keir et al., 2016; Krustrup et al., 2004; Poole et al., 1991; Russell et al., 2002; Zoladz et al., 2016). This additional O_2_ cost (i.e., the slow component of O_2_ uptake kinetics, ${\dot{V}}_{O_{2}\mathrm{sc}}$) reflects reduced work efficiency and appears to supplement the fundamental kinetics observed during moderate intensity exercise (Rossiter, 2011). In the past, Poole and colleagues (Poole et al., 1991, 1988) demonstrated that the mechanism explaining the ${\dot{V}}_{O_{2}\mathrm{sc}}$ is likely to reside in the biochemical processes of exercising muscle. During the past years, a body of evidence has been growing to link the origin of ${\dot{V}}_{O_{2}\mathrm{sc}}$ to the oxidative and glycolytic ATP turnover in the myocytes (Colosio et al., 2020; Korzeniewski & Rossiter, 2020; O'Connell et al., 2017; Zoladz et al., 2016). A contribution by the respiratory muscles to the O_2_ cost cannot be rejected (Cross et al., 2014; O'Connell et al., 2017), but much of the oxidative inefficiency occurs as a consequence of disturbance inside the myofibres. Colosio et al. (2020) raised the hypothesis that the ${\dot{V}}_{O_{2}\mathrm{sc}}$ could be explained by an increased contribution of aerobic metabolism to ATP resynthesis, mirrored by a decreased contribution of anaerobic ATP resynthesis over time in agreement with the theory of Korzeniewski and Rossiter (2015).

Zoladz et al. (2016) state indeed that the ${\dot{V}}_{O_{2}\mathrm{sc}}$ occurring in the working muscle can be potentially caused by a decreased efficiency on the side of ATP production (decrease in the power output ratio) and/or on the side of ATP consumption (increase in the ATP usage/power output ratio). In this context, muscle fatigue and the recruitment of less efficient muscle fibres are considered the primary mechanisms explaining the ${\dot{V}}_{O_{2}\mathrm{sc}}$ (Cannon et al., 2011; Grassi et al., 2015; Keir et al., 2016). Given the theory of Zoladz et al. (2016), measurements of muscle ${\dot{V}}_{O_{2}}$ ($m{\dot{V}}_{O_{2}}$) kinetics (muscle oxidative capacity, O_2_ uptake rate in the muscle) using the repeated ischaemia approach with near‐infrared spectroscopy measurements (Zuccarelli et al., 2020) would be useful to test this hypothesis in a ‘real’ context. Furthermore, an increase in the deoxygenated haemoglobin (HHb) variable could indicate changes in the ratio between $m{\dot{V}}_{O_{2}}$ and O_2_ delivery in the investigated tissue. Taken together, the analysis of both these variables could be useful for a wider understanding of muscle oxidative activity during cycling exercise.

A temporal association between the ${\dot{V}}_{O_{2}}$ kinetics (${\dot{V}}_{O_{2}\mathrm{sc}}$ and time constant τ) and the time course of peripheral muscle fatigue during high‐intensity exercise has been suggested (Keir et al., 2016; Temesi et al., 2017). However, other authors have demonstrated contrary evidence regarding the relationship between ${\dot{V}}_{O_{2}}$ kinetics and locomotor muscle fatigue (de Souza et al., 2016; Hopker et al., 2016; Thistlethwaite et al., 2008). Do Nascimento Salvador et al. (2018, 2019) have shown that changes in ${\dot{V}}_{O_{2}\mathrm{sc}}$ and/or τ did not reflect alterations in muscle force production and fatigue during very heavy cycling in healthy male and female subjects.

The main purpose of this study was to explain the ${\dot{V}}_{O_{2}\mathrm{sc}}$ and muscle fatigue during cycling in different exercise intensity domains. The secondary purpose was to verify the physiological mechanisms associated with the ${\dot{V}}_{O_{2}\mathrm{sc}}$ phenomenon. It was hypothesised that (1) muscle fatigue would be related to muscle oxidative capacity, HHb kinetics and EMG activity, (2) ${\dot{V}}_{O_{2}\mathrm{sc}}$ would be associated with muscle activation and recruitment, and (3) muscle fatigue per se cannot explain the ${\dot{V}}_{O_{2}\mathrm{sc}}$ phenomenon.

## 2 METHODS

### 2.1 Ethical approval

The study was approved by the Research Ethics Committee of the Federal University of Santa Catarina (approval no. 3.496.051) and was conducted in accordance with the *Declaration of Helsinki* except for registration in a database. After being fully informed of the risks and stresses associated with the study, the subjects gave their written informed consent to participate.

### 2.2 Participants

Sample size was calculated a priori based on effect size of 0.5 (moderate effect) and *P* = 0.05, set at a minimum power of 80% of the statistical analysis. Based on these parameters, a minimum of 12 participants were required. Thus, 16 healthy physically active males (age 28 ± 5 years; mass 75.1 ± 7.1 kg; height 175.8 ± 5.8 cm; sum of skinfolds (triceps, supra‐iliac, abdominal and mid‐thigh) 56.4 ± 19.3 mm) volunteered to participate in the study. They participated in any exercise at a recreational level (3–4 sessions per week; at least 150 min per week) and were familiar with laboratory exercise testing procedures.

### 2.3 Overview of study design

**FIGURE 1:**
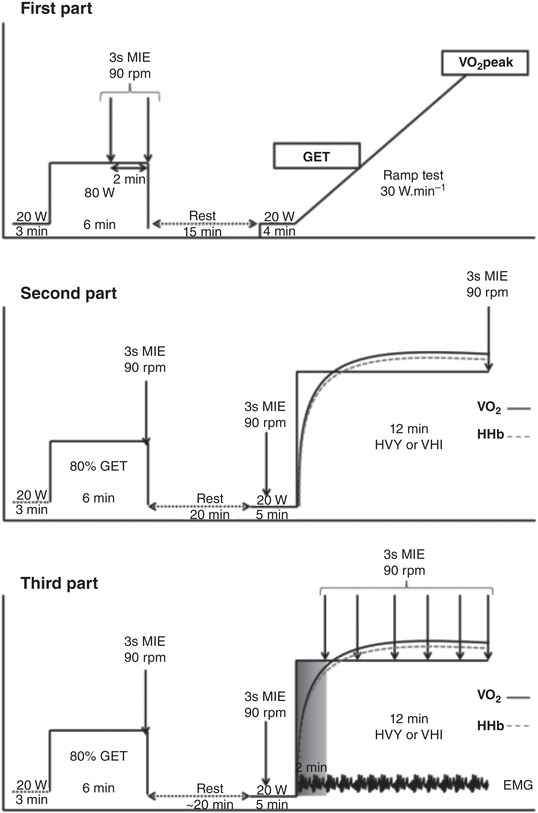
Experimental design of the study. GET, gas exchange threshold; HHb, deoxygenated haemoglobin value; MIE, maximal isokinetic effort; HVY, heavy exercise; VHI, very heavy intensity exercise; ${\dot{V}}_{O_{2}\mathrm{peak}}$, the highest ${\dot{V}}_{O_{2}}$ value obtained in a 15‐s interval during ramp test.

The experimental protocol was composed of seven visits to the laboratory at the same time of day in a controlled environmental laboratory condition (∼22°C; 50–60% relative humidity) and separated by at least 24 h in a period of 1 month. Subjects were instructed to avoid any intake of caffeine for 3 h, or alcohol and strenuous exercise in the 24 h preceding the test sessions and to arrive at the laboratory in a rested and fully hydrated state, at least 2 h postprandial. The study was divided into three parts. On the first visit, after a familiarization with the equipment and protocols and after anthropometrics measurements, a muscle oxidative capacity test was conducted. After this initial part, each subject performed a maximal incremental ramp test for the determination of the GET, ${\dot{V}}_{O_{2}\mathrm{peak}}$ and peak power output. In the second part, subjects performed constant work rate bouts at moderate (MOD) followed by heavy (HVY) or very heavy intensity (VHI) exercise. Finally, in the third part, two additional constant work rate bouts for HVY or VHI exercise were completed with measurement of muscle force production during exercise (Figure 1). Breath‐by‐breath pulmonary gas exchange, heart rate (HR) data and HHb changes were measured continuously during all tests.

### 2.4 Equipment

All tests were performed on an electromagnetically braked cycle ergometer instrumented with pedal force measurement (Excalibur Sport PFM, Lode BV, Groningen, the Netherlands). Capillary blood samples (25 μl) were obtained from the earlobe of each subject before, during (at mid‐time) and after exercise to measure the blood lactate concentration ([La]) using an electrochemical analyser (YSI 2700 Stat, YSI Life Sciences, Yellow Springs, OH, USA). Respiratory and pulmonary gas exchange variables were measured using a breath‐by‐breath analyser (Quark PFTergo, Cosmed, Rome, Italy). Before each test, the O_2_ and CO_2_ analysis systems were calibrated using ambient air (20.94% O_2_ and 0.03% CO_2_) and a gas of known O_2_ and CO_2_ concentration (16.00% O_2_ and 5.00% CO_2_) according to the manufacturer's instructions. Likewise, the turbine flow meter was calibrated before each test using a 3‐litre syringe (Quark PFTergo). A wristwatch cardiac monitor (Polar S810, Polar Electro Oy, Kempele, Finland) was used to measure the HR. The cycle ergometer and the electrochemical analyser were also calibrated in accordance with specific manufacturer's recommended procedures. A portable continuous‐wave, spatially resolved near‐infrared (NIRS) light photometer (PortaLite, Artinis Medical Systems, Elst, the Netherlands) was utilized to measure the oxygenation changes in the right limb. Surface electromyogram (EMG) activity of right lower limb muscles was recorded from the muscle bellies of rectus femoris (RF), biceps femoris long head (BF) and vastus lateralis (VL) with a common mode rejection ratio of 110 dB and an input impedance of 10 GΩ with a four‐channel EMG system (New Miotool 800 USB, Miotec, Porto Alegre, Brazil) in the third part of the study. Rating of perceived exertion (RPE) was obtained according to a Borg scale (6–20 points).

### 2.5 Determination of GET and ${\dot{V}}_{O_{2}\mathrm{peak}}$

After a 4‐min period of cycling at 20 W (baseline), an incremental ramp test to exhaustion was undertaken with power output increasing at a rate of 30 W/min. Values of RPE were recorded every 2 min. Subjects were instructed to maintain their preferred cadence (70–80 rpm) throughout the test. The preferred cadence along with saddle and handlebar height and configuration was recorded and reproduced in subsequent tests. Each subject was verbally encouraged to undertake maximal effort. The test was terminated when the cadence fell by more than 10 rpm below the preferred cadence for more than 5 s despite strong verbal encouragement (Black et al., 2015). The ${\dot{V}}_{O_{2}}$ values were averaged over 15‐s periods. ${\dot{V}}_{O_{2}\mathrm{peak}}$ was defined as the highest value obtained in a 15‐s interval. Since, ${\dot{V}}_{O_{2}\max}$ confirmation was not the study's focus, to prevent discussion about successful or unsuccessful ${\dot{V}}_{O_{2}\max}$ attainment, ${\dot{V}}_{O_{2}\mathrm{peak}}$ terminology was used. This terminology was criticized by Poole and Jones (2017), but the use of another maximum session to confirm the ${\dot{V}}_{O_{2}\mathrm{peak}}$ values was unsuitable in the study design. Considering that the primary criterion for the analysis of VO_2peak_ was the attainment of a plateau in oxygen uptake, despite an increased workload, according to the methodology of Day et al. (2003). The secondary criteria, [La] higher than 8.0 mmol/l and a respiratory exchange ratio >1.10, were also used as criteria for ${\dot{V}}_{O_{2}\mathrm{peak}}$ achievement. The peak power output was considered as the highest power output attained during the test. The GET was determined using a cluster of measurements as the V‐slope method and the ventilatory equivalent method (Beaver et al., 1986). The data from the ramp test were used to calculate the work rate corresponding to HVY or VHI exercise (i.e., GET plus 30% or 60% of the difference between the work rate at the GET and ${\dot{V}}_{O_{2}\mathrm{peak}}$).

### 2.6 Tissue oxygenation measurements

Oxygenation changes in a superficial portion of VL muscles of right legs were evaluated by NIRS. The instruments provide measurements of micromolar changes in oxygenated and deoxygenated haemoglobin and myoglobin concentrations (i.e., O_2_Hb and HHb, respectively), or tissue saturation index (TSI). The NIRS probe consists of three light transmitters (each emitted two wavelengths of 760 and 850 nm) separated by 3, 3.5 and 4 cm from the receiving optode. The deepest signal (4 cm) was taken into account for the analysis. Thus, the light penetration depth can be estimated to be at least 2 cm. Skinfold thicknesses at the sites of probes application were determined (9.2 ± 4.5 mm) and were less than 1 cm. The skin was carefully shaven prior to the experimentation. The probes were firmly attached to the skin overlying the lower third of vastus lateralis muscles (∼10 cm above the knee joint) of the right limbs, parallel to the major axis of the thigh, by elastic medical bands and by adhesive tape. The place where the probes were attached was recorded using a skin marker and reproduced throughout the tests. The sampling frequency was set at 10 Hz. HHb values with respect to an initial value arbitrarily set equal to zero were calculated and expressed as a percentage of the physiological normalization values obtained before the exercise. An ischaemic/hyperaemia calibration of the right limb (i.e., physiological normalization) was made before each visit to normalize HHb values according to previous literature (McCully et al., 1994).

### 2.7 Muscle oxidative capacity test

Following the method proposed by Ryan et al. (2012) and Adami and Rossiter (2018), muscle ${\dot{V}}_{O_{2}}$ ($m{\dot{V}}_{O_{2}}$) was estimated by calculating the slope of the initial linear increase in HHb during short (5–10 s) bouts of ischaemia induced by rapid inflation and deflation of a pneumatic cuff during the recovery from short leg extension exercise (<1 min). The intermittent arterial occlusion protocol and the fit procedures were published elsewhere in our previous study (Zuccarelli et al., 2020).

### 2.8 Constant work rate bouts and maximal isokinetic effort

In the second part of this investigation (Figure 1), two constant work rate bouts of 12 min were performed for HVY and two constant work rate bouts were performed for VHI exercise in a randomized order. Each visit started with 3 min of baseline (cycling at 20 W) followed by an abrupt transition to moderate exercise (80% of GET) during 6 min immediately followed by the maximal isokinetic effort (MIE) in the seated position, that is, 3 s at 90 rpm. After 4 min of active recovery, a passive recovery was set until baseline values of main variables (i.e., ${\dot{V}}_{O_{2}}$, HR, HHb) were reached again. Then, 2 min of baseline cycling was followed by a MIE of 3 s, 3 min of 20 W cycling and an abrupt transition to the target work rate for HVY or VHI exercise for 12 min. Capillary blood samples were collected from the ear lobe 30 s before the beginning, as well as 30 s before the sixth minute, and 30 s before the end of constant work rate bouts. A MIE was repeated at the very end of each constant work rate exercise (Figure 1). In the third part, the MIE was performed every 2 min during HVY or VHI bouts.

For the muscle fatigue measurements, the torque and power data were recorded continuously during all the MIE. The peak torque during the MIE was then considered as the average of the peak values of both left and right crank arms using a visual inspection considering a minimum window of 2 s and excluding outlier values. The MIE protocol and the analysis procedures were published elsewhere in our previous study (do Nascimento Salvador et al., 2018, 2019).

### 2.9 EMG measurements

In the third part of this study, EMG signals were recorded from the right quadriceps during the entire bout using bipolar 20‐mm‐diameter Ag–AgCl surface electrodes with full‐surface solid adhesive hydrogel. The skin over the muscle was shaved, sandpapered and cleaned. The reference electrode was placed on the tibia bone medial surface, and other electrodes were placed in the approximate direction of muscle fibres of VL, RF and BF. The position of each electrode was recorded and repeated in the same location at subsequent visits. The EMG signals were amplified resulting in a sample rate of 2000 Hz. A band‐pass filter of 20–500 Hz was applied. A Butterworth filter of 5° was used. The data were then rectified and smoothed using root‐mean‐square (RMS) analysis. Windows of 3 s were applied in every MIE performed and RMS mean values were calculated. Fast Fourier transformation was applied to verify the frequency spectrum behaviour distribution excluding the higher and lower band and dividing into five bands (B1, B2, B3, B4 and B5) between 20 and 600 Hz. Both the two higher (>100 Hz; B4 + B5) and the two lower (<100 Hz; B1 + B2) bands were grouped for analysis, signalling the recruitment of higher‐threshold motor units (HTMUs) and lower‐threshold motor units (LTMUs), respectively. The middle band (B3) was excluded. Mean power frequency (MPF) was determined for each 3 s window in every MIE. Besides, RMS and MPF were calculated for 30 s windows in every minute of exercise avoiding the MIE period.

### 2.10 Data analysis

Breath‐by‐breath data were processed and analysed as described previously (do Nascimento Salvador et al., 2018). In short, each condition was initially examined to exclude any outlying values (caused by sighs, swallowing and coughs), linearly interpolated to 1‐s intervals, time‐aligned to the start of exercise, and ensemble averaged to yield a single profile for each participant. The single profile data were reduced to a 5 s stationary average. The first 20 s of data after the onset of exercise (i.e., the ‘cardiodynamic’ phase) were not included in the analysis. Non‐linear regression techniques were used to fit the data after the onset of a fundamental phase with an exponential function (Equation 1). An iterative process ensured that the sum of squared errors was minimized (OriginPro 8; OriginLab Corp., Northampton, MA, USA). Based on a previous study (Barstow et al., 1996), the model was constrained in ${\dot{V}}_{O_{2}\mathrm{baseline}}$ to aid in the identification of the key parameters according to the following equation:

(1) $${\dot{V}}_{O_{2}}\left(t\right)={\dot{V}}_{O_{2}\mathrm{baseline}}+A\times \;\left[1-e^{-\left(\frac{t-\mathrm{TD}}{\tau }\right)}\right]$$

where ${\dot{V}}_{O_{2}}$(*t*) represents the value of ${\dot{V}}_{O_{2}}$ at a given time (*t*); ${\dot{V}}_{O_{2}\mathrm{baseline}}$ is the average value over the last minute of baseline cycling; *A* is the asymptotic amplitude for the exponential term describing changes in ${\dot{V}}_{O_{2}}$ from baseline to its asymptote; τ is the time constant; and the TD is the time delay.

The fundamental phase was isolated following the iterative method to identify the exponential region by considering the criteria proposed in the literature (Murgatroyd et al., 2011; Rossiter et al., 2002, 2001). The identification of ${\dot{V}}_{O_{2}\mathrm{sc}}$ during HVY or VHI bouts, in which the exponential region varies in duration among the participants due to the variably delayed appearance of the ${\dot{V}}_{O_{2}\mathrm{sc}}$ (Murgatroyd et al., 2011), was performed individually. The identification at the end of fundamental phase (i.e., time delay of slow phase = TD_s_) was performed by fitting a window from the start of the fundamental phase and lengthening iteratively until the exponential model fit demonstrated a discernible and consistent departure from the measured ${\dot{V}}_{O_{2}}$ values by considering the criteria proposed (Rossiter et al., 2001) and described previously (do Nascimento Salvador et al., 2018, 2019). ${\dot{V}}_{O_{2}\mathrm{sc}}$ was calculated according to the following equation:

(2) $${\dot{V}}_{O_{2}\mathrm{sc}}={\dot{V}}_{O_{2}\mathrm{end}}-\left({\dot{V}}_{O_{2}\mathrm{baseline}}+A\right)$$

where, ${\dot{V}}_{O_{2}\mathrm{end}}$ is the average ${\dot{V}}_{O_{2}}$ value over the last 30 s at 12 min of exercise. HHb kinetics have been analysed applying Equation (1) for ${\dot{V}}_{O_{2}}$. The HHb values in each transition for each condition were normalized by physiological normalization, ensemble averaged and reduced to a 5‐s stationary average to perform the exponential fit.

### 2.11 Statistical analysis

Descriptive statistics are expressed as means ± standard deviation. The Shapiro–Wilk test was applied to ensure a Gaussian distribution of the data (*n* < 50). Relationships between variables were assessed using Pearson's product‐moment correlation coefficient. Simple and multiple linear regression was used to identify the power of a variables in explaining one another. Student's paired *t*‐test was used to compare differences between two variables or the Wilcoxon test was used for non‐parametric analyses (i.e., TD_s_). A one‐way ANOVA with repeated measures was used to analyse the difference between conditions. In some cases, a two‐way ANOVA was used to identify the interaction condition vs. time. Assumptions of sphericity were assessed using Mauchly's test, and any violations were corrected using the Greenhouse–Geisser correction factor. The Shapiro–Wilk test was used to verify the normality of residuals. When significant main effects were observed, Tukey's *post hoc* test was used for comparisons. Analyses were performed using the GraphPad Prism software package for Windows (version 7.0; GraphPad Software Inc., San Diego, CA, USA). The level of significance was set at *P* < 0.05.

## 3 RESULTS

During the incremental test, the GET was attained at 131 ± 35 W and represented 59 ± 5% of ${\dot{V}}_{O_{2}\mathrm{peak}}$. The respiratory compensation point (RCP) was 222 ± 39 W achieved at 80 ± 6% of ${\dot{V}}_{O_{2}\mathrm{peak}}$. The peak power output, maximal HR values, blood [La] peak values ([La]_peak_) and ${\dot{V}}_{O_{2}\mathrm{peak}}$ were 325 ± 49 W, 179 ± 11 bpm, 11.3 ± 3.0 mmol/l and 3.4 ± 0.6 l/min (44.6 ± 6.6 ml/kg/min), respectively. Just 3 of the 16 participants did not attain a plateau in the ${\dot{V}}_{O_{2}}$ final values, but all of them achieved the secondary criteria for ${\dot{V}}_{O_{2}\mathrm{peak}}$ determination.

### 3.1 ${\dot{V}}_{O_{2}}$ kinetics

**FIGURE 2:**
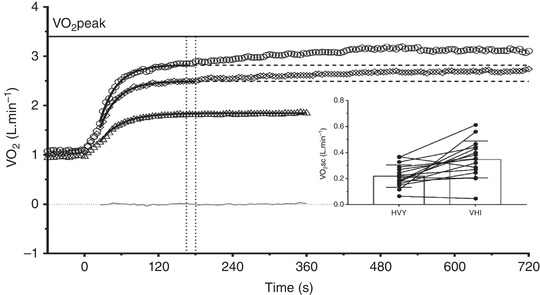
Mean values of ${\dot{V}}_{O_{2}}$ kinetics during moderate (triangles), heavy (HVY, rhombus) and very heavy intensity (VHI, circles) exercise. Vertical dotted lines indicate the start of ${\dot{V}}_{O_{2}}$ slow component (${\dot{V}}_{O_{2}\mathrm{sc}}$) and horizontal dashed lines indicate the ${\dot{V}}_{O_{2}}$ asymptote projection. Bold lines show the exponential curves and grey line shows the residual mean values. The inside figure shows the individual values of ${\dot{V}}_{O_{2}\mathrm{sc}}$ during HVY and VHI conditions (${\dot{V}}_{O_{2}\mathrm{sc}}$, *t* = 3.83; *P* = 0.002). Note that the peak values of ${\dot{V}}_{O_{2}}$ are not achieved during any condition; *n* = 16 participants.

The HVY and VHI constant work rate bouts were performed at 176 ± 36 and 221 ± 39 W and represented 54 ± 5% and 67 ± 5% of peak power output, respectively. The two intensities were performed at 71 ± 4% and 84 ± 2% of ${\dot{V}}_{O_{2}\mathrm{peak}}$, respectively. The [La] at the end of HVY and VHI constant work rate bouts was 5.7 ± 2.3 and 11.2 ± 3.3 mmol/l, respectively. The final HR values for HVY and VHI exercise represented 87 ± 6% and 98 ± 4% of maximal HR values attained at ramp test, respectively. These intensities achieved a RPE of 14 ± 2 and 18 ± 2, respectively. During VHI exercise, *A* (*F* = 154; *P* < 0.0001), *A*_total_ (i.e., *A* + ${\dot{V}}_{O_{2}\mathrm{baseline}}$) (*F* = 203; *P* < 0.0001), ${\dot{V}}_{O_{2}\mathrm{sc}}$ (*t* = 3.83; *P* = 0.002) and ${\dot{V}}_{O_{2}\mathrm{end}}$ (*F* = 275; *P* < 0.0001) were significantly higher compared to HVY and MOD exercise (Table 1 and Figure 2). A significant relationship between ${\dot{V}}_{O_{2}\mathrm{sc}}$ and ∆[La] was observed independently of exercise intensity (*R* ^2^ = 0.42; *F* = 17.7; *P* = 0.001).

**TABLE 1:** ${\dot{V}}_{O_{2}}$ kinetics variables obtained during constant work rate bouts in the moderate (MOD), heavy (HVY) and very heavy intensity (VHI) domains.

	${\dot{V}}_{O_{2}\mathrm{baseline}}$ (l/min)	*A* (l/min)	*A*_total_ (l/min)	${\dot{V}}_{O_{2}\mathrm{sc}}$ (l/min)	${\dot{V}}_{O_{2}\mathrm{sc}}$% (%${\dot{V}}_{O_{2}\mathrm{res}}$)	TD (s)	τ (s)	TD_s_ (s)	${\dot{V}}_{O_{2}\mathrm{end}}$ (l/min)
MOD	0.99 ± 0.11 (0.9–1.0)^a^	0.86 ± 0.28 (0.7–1.0)^a^	1.84 ± 0.31 (1.7–2.0)^a^			12.4 ± 5.2 (9.6–15.1)	34.1 ± 9.0 (29.2–38.9)^a^		1.87 ± 0.32 (1.7–2.0)^a^
HVY	1.05 ± 0.10 (1.0–1.1)^b^	1.44 ± 0.35 (1.3–1.6)^b^	2.49 ± 0.4 (2.3–2.7)^b^	0.22 ± 0.09 (0.17–0.26)^a^	28 ± 12 (21–34)^a^	14.3 ± 2.4 (13.0–15.6)	28.3 ± 7.1 (24.5–32.1)^a,b^	179 ± 16 (170–188)	2.71 ± 0.43 (2.5–2.9)^b^
VHI	1.09 ± 0.09 (1.0–1.1)^b^	1.71 ± 0.42 (1.5–1.9)^c^	2.79 ± 0.46 (2.6–3.0)^c^	0.35 ± 0.14 (0.27–0.42)^b^	69 ± 28 (54–84)^b^	15.1 ± 3.9 (13.0–17.1)	26.0 ± 5.0 (23.3–28.6)^b^	165 ± 25 (152–178)	3.14 ± 0.50 (2.88–3.41)^c^

Values are means ± SD (95% CI). Different letters denote statistical differences, *P* < 0.05. There were significant differences between intensities for ${\dot{V}}_{O_{2}\mathrm{baseline}}$, lower at MOD vs. HVY, *P* = 0.001 and vs. VHI, *P* = 0.001; *A* and *A*_total_, higher for VHI vs. MOD, *P* < 0.0001 and vs. HVY, *P* < 0.0001 and HVY vs. MOD, *P* < 0.0001; ${\dot{V}}_{O_{2}\mathrm{sc}}$ and %${\dot{V}}_{O_{2}\mathrm{res}}$, higher for VHI vs. HVY, *P* = 0.002 and *P* < 0.0001, respectively; TD, *F* = 3.04, *P* = 0.072; τ, lower for VHI vs. MOD, *P* = 0.013; TD_s_ *P* = 0.072; ${\dot{V}}_{O_{2}\mathrm{end}}$, higher for VHI vs. MOD, *P* < 0.0001 and vs. HVY, *P* < 0.0001 and HVY vs. MOD, *P* < 0.0001; *n* = 16 participants. *A*, fundamental amplitude; *A*_total_, amplitude + ${\dot{V}}_{O_{2}\mathrm{baseline}}$; τ, time constant; TD, time delay; TD_s_, time delay of slow phase; %${\dot{V}}_{O_{2}\mathrm{res}}$, reserve ${\dot{V}}_{O_{2}}$; ${\dot{V}}_{O_{2}\mathrm{baseline}}$, mean of ${\dot{V}}_{O_{2}}$ in the last minute of baseline cycling; ${\dot{V}}_{O_{2}\mathrm{end}}$, mean ${\dot{V}}_{O_{2}}$ in the last 30 s of exercise; ${\dot{V}}_{O_{2}\mathrm{sc}}$, slow component of ${\dot{V}}_{O_{2}}$.

**FIGURE 3:**
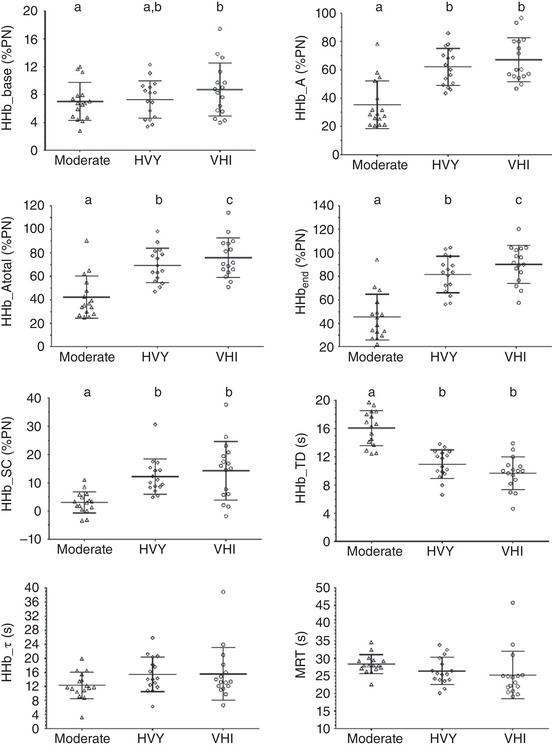
Individual values in each variable for HHb kinetics during heavy (HVY) and very‐heavy intensity (VHI) exercise. Different letters denote statistical differences, *P* < 0.05. There were significant differences between intensities for HHb_base, higher for VHI vs. MOD, *P* = 0.026; HHb_*A*, lower at MOD vs. HVY *P* < 0.0001 and vs. VHI, *P* < 0.0001; HHb_*A*_total_, higher for VHI vs. MOD, *P* < 0.0001 and vs. HVY, *P* = 0.025 and HVY vs. MOD, *P* < 0.0001; HHb_SC, lower at MOD vs. HVY, *P* = 0.001 and vs. VHI, *P* = 0.001; HHb_end_, higher for VHI vs. MOD, *P* < 0.0001 and vs. HVY, *P* = 0.029 and HVY vs. MOD, *P* < 0.0001; HHb_TD, lower at MOD vs. HVY, *P* < 0.0001 and vs. VHI, *P* < 0.0001. HHb_τ (*F* = 2.98, *P* = 0.093) and MRT (*F* = 3.13, *P* = 0.088) were not significantly different between intensities; *n* = 16 participants. HHb, deoxygenated haemoglobin; HHb_base, mean of the last minute of baseline cycling; HHb_*A*, fundamental amplitude; HHb_*A*_total_, HHb_*A* + HHb_base; HHb_end, mean of the last 20 s of exercise; HHb_SC, slow component of HHb (subtraction of HHb_end_ ‐ HHb_*A*_total_); HHb_τ, time constant; HHb_TD, time delay; MRT, mean response time (TD + τ); PN, physiological normalization.

In relation to HHb kinetics, the total amplitude of the fundamental phase (HHb_*A*_total_; *F* = 91.9; *P* < 0.0001) and the final values obtained (HHb_end_; *F* = 91.0; *P* < 0.0001) increased as a dose–response for intensity (they were higher for VHI vs. HVI, VHI vs. MOD, HVI vs. MOD; Table 2). It stands out that despite a different time delay (HHb_TD) between the moderate and the other intensities (*F* = 65.9; *P* < 0.0001), there were no significant differences for HHb_τ (*F* = 2.98; *P* = 0.093) or for mean response time (MRT; TD + τ; *F* = 3.13; *P* = 0.088). It is noteworthy that HHb_SC was not significantly different between HVY and VHI constant work rate bouts (*P* = 0.752; see details in Table 2; Figure 3).

**TABLE 2:** Kinetics variables of the deoxygenated haemoglobin (HHb) obtained during constant work rate bouts in the moderate (MOD), heavy (HVY) and very heavy intensity (VHI) domains.

	HHb_base (%PN)	HHb_*A* (%PN)	HHb_*A*_total_ (%PN)	HHb_SC (%PN)	HHb_end_ (%PN)	HHb_TD (s)	HHb_τ (s)	MRT (s)	*R* ^2^
MOD	7.0 ± 2.7 (6–8)^a^	35.3 ± 16.9 (26–44)^a^	42.3 ± 17.9 (33–52)^a^	3.0 ± 3.8 (1–5)^a^	45.4 ± 19.4 (35–56)^a^	16.1 ± 2.5 (15–17)^a^	12.3 ± 3.8 (10–14)	28.4 ± 2.7 (27–30)	0.94 ± 0.03 (0.93–0.96)
HVY	7.3 ± 2.7 (6–9)^a,b^	62.0 ± 13.1 (55–69)^b^	69.3 ± 14.6 (62–77)^b^	12.2 ± 6.3 (9–16)^b^	81.6 ± 15.5 (73–90)^b^	11.0 ± 2.0 (10–12)^b^	15.4 ± 5.0 (13–18)	26.4 ± 3.9 (24–29)	0.97 ± 0.02 (0.96–0.98)
VHI	8.7 ± 3.8 (7–11)^b^	67.1 ± 15.6 (59–76)^b^	75.9 ± 16.9 (67–85)^c^	14.3 ± 10.4 (9–20)^b^	90.2 ± 16.1 (82–99)^c^	9.7 ± 2.3 (8–11)^b^	15.6 ± 7.5 (12–20)	25.3 ± 6.7 (22–29)	0.97 ± 0.03 (0.95–0.99)

Values are means ± SD (95% CI). Different letters denote statistical differences, *P* < 0.05. There were significant differences between intensities for HHb_base, higher for VHI vs. MOD, *P* = 0.026; HHb_*A*, lower at MOD vs. HVY, *P* < 0.0001 and vs. VHI, *P* < 0.0001; HHb_*A*_total_, higher for VHI vs. MOD, *P* < 0.0001 and vs. HVY, *P* = 0.025 and HVY vs. MOD, *P* < 0.0001; HHb_SC, lower at MOD vs. HVY, *P* = 0.001 and vs. VHI, *P* = 0.001; HHb_end_, higher for VHI vs. MOD, *P* < 0.0001 and vs. HVY, *P* = 0.029 and HVY vs. MOD, *P* < 0.0001; HHb_TD, lower at MOD vs. HVY, *P* < 0.0001 and vs. VHI, *P* < 0.0001. HHb_τ (*F* = 2.98, *P* = 0.093) and MRT (*F* = 3.13, *P* = 0.088) were not significantly different between intensities; *n* = 16 participants. HHb, deoxygenated haemoglobin; HHb_*A*, fundamental amplitude; HHb_*A*_total_, HHb_*A* + HHb_base; HHb_base, mean of the last minute of baseline cycling; HHb_end, mean of the last 20 s of exercise; HHb_SC, slow component of HHb (subtraction of HHb_end_ – HHb_*A*_total_); HHb_τ, time constant; HHb_TD, time delay; MRT, mean response time (TD + τ); PN, physiological normalization.

### 3.2 Torque production

**FIGURE 4:**
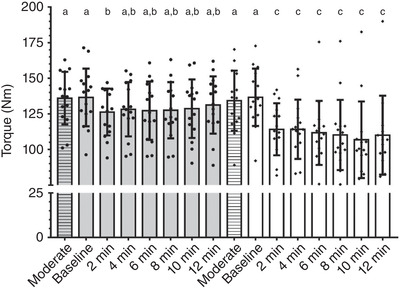
Individual values in each moment for torque production during moderate (dashed bars), heavy (grey bars) and very‐heavy intensity (white bars) exercise in the third part of this study. Bars indicate mean values and error bars indicate standard deviation. Different letters denote statistical differences, *P* < 0.05. There was a significant condition vs. time interaction (*F* = 8.29; *P* < 0.0001). There was also a significant main effect between conditions (*F* = 22.43; *P* < 0.0001) and time (*F* = 15.47; *P* < 0.0001); *n* = 16 participants.

There were no significant differences for torque production between moderate domain and baseline period before the main exercise (*F* = 0.041; *P* > 0.999). In the same way, significant differences between visits for both HVY (*F* = 0.434; *P* = 0.598) and VHI (*F* = 0.110; *P* = 0.883) exercise were not observed within each condition. When torque was compared among conditions MOD, HVY and VHI exercise in the second part, there was a significant condition versus time effect (*F* = 44.45; *P* < 0.0001). In other words, in all visits, muscle fatigue in VHI exercise was higher compared with HVY and MOD exercise. Nonetheless, there was no significant difference between HVY 12 min and MOD during the third part (*P* = 0.924; see details in Table 3 and Figure 4). There were no significant differences between ∆torque 2 min (12.5 ± 9.5 N m) and ∆torque 12 min (7.7 ± 10.4 N m) for HVY (*P* = 0.597) and VHI (∆torque 2 min 23.3 ± 9.5 N m, ∆torque 12 min 30.0 ± 24.9 N m; *P* = 0.944), demonstrating that the greatest loss in the force production occurred during the beginning of the exercise in which there is no ${\dot{V}}_{O_{2}\mathrm{sc}}$. The ∆torque 2 min presented a significant correlation with HHb_end_ in VHI exercise (*r* = −0.522, *P* = 0.046). The ∆torque 12 min during HVY and VHI was correlated with lower and higher MPF bands in RF isolated muscle (B1 + B2, *R* ^2^ = 0.30; *F* = 8.41; *P* = 0.009; B4 + B5, *R* ^2^ = 0.31; *F* = 8.82; *P* = 0.008). When all muscles were analysed together, just higher MPF bands demonstrated a relationship with ∆torque 12 min (B1 + B2, *R* ^2^ = 0.04; *F* = 0.83; *P* = 0.372; B4 + B5, *R* ^2^ = 0.27; *F* = 7.31; *P* = 0.014). A significant relationship was found between ∆torque 12 min and ∆[La] independently of exercise intensity (*R* ^2^ = 0.652; *F* = 45.0; *P* < 0.0001).

**TABLE 3:** Torque results (N m) obtained during constant work rate bouts in the moderate (MOD), heavy (HVY) and very heavy intensity (VHI) domains.

		MOD	Baseline	2 min	4 min	6 min	8 min	10 min	12 min
Second part	HVY	136.0 ± 21.5 (125–148)^a^	136.8 ± 22.6 (125–149)^a^						127.4 ± 23.4 (115–140)^b^
	VHI	136.2 ± 20.7 (125–147)^a^	137.3 ± 20.2 (127–148)^a^						104.2 ± 23.4 (92–117)^c^
Third part	HVY	136.1 ± 18.4 (126–146)^a^	136.6 ± 20.2 (125–148)^a^	126.4 ± 16.4 (117–135)^b^	128.3 ± 19.0 (118–139)^a,b^	127.2 ± 20.5 (116–139)^a,b^	127.6 ± 19.7 (117–139)^a,b^	128.7 ± 20.5 (117–140)^a,b^	131.2 ± 20.1 (120–142)^a,b^
	VHI	134.2 ± 21.2 (123–146)^a^	136.7 ± 20.1 (126–148)^a^	114.2 ± 18.3 (104–124)^c^	114.2 ± 20.8 (103–126)^c^	111.7 ± 22.5 (99–124)^c^	110.2 ± 24.6 (97–124)^c^	106.9 ± 26.9 (90–124)^c^	110.1 ± 27.7 (93–127)^c^

Values are means ± SD (95% CI). Different letters denote statistical differences, *P* < 0.05. There was a significant condition vs. time interaction: *F* = 8.29; *P* < 0.0001; *n* = 16 participants.

### 3.3 EMG

The EMG activity was completely analysed in 11 participants. Five participants were excluded from the EMG analysis because of problems with the signal or data collection during visits. During the MIE the RMS signal was not significantly different between conditions (condition, *F* = 0.71, *P* = 0.402; interaction, *F* = 0.03, *P* > 0.999). This indicates that muscle activation during the MIE was independent of exercise intensity. Moreover, there was no effect on intensity or time for different frequency bands in MPF (B1 + B2 condition, *F* = 1.06, *P* = 0.305; time, *F* = 0.15, *P* = 0.988; interaction, *F* = 0.11, *P* = 0.996; B4 + B5 condition, *F* = 1.82, *P* = 0.179; time, *F* = 0.44, *P* = 0.850; interaction, *F* = 0.02, *P* > 0.999). This is evidence that during MIE the pattern of muscle activation is the same independent of exercise intensity.

**FIGURE 5:**
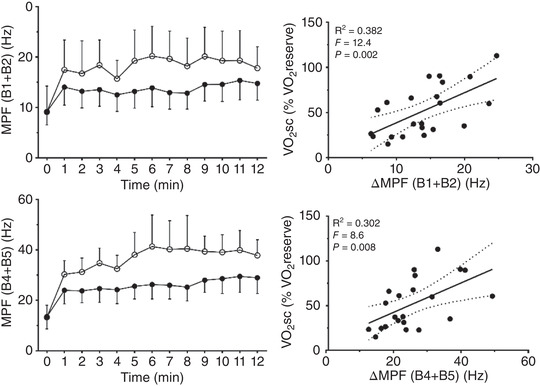
Left upper and lower panels: mean power frequency (MPF) of electromyogram activity of rectus femoris during heavy (black circles) and very heavy intensity (open circles) exercise. In both panels there was a significant effect of time (B1 + B2, *F* = 2.21, *P* = 0.011; B4 + B5, *F* = 6.63, *P* < 0.0001) and of condition (B1 + B2, *F* = 29.4, *P* < 0.0001; B4 + B5, *F* = 66.7, *P* < 0.0001) but there was no interaction (B1 + B2, *F* = 0.39, *P* = 0.966; B4 + B5, *F* = 0.89, *P* = 0.556). Right panels: relationship between ${\dot{V}}_{O_{2}}$ slow component (${\dot{V}}_{O_{2}\mathrm{sc}}$) and different MPF bands in all muscles grouped. ∆MPF, difference between 12 min and zero). Upper panel shows lower bands (B1 + B2) and lower panel shows higher bands (B4 + B5); *n* = 11 participants.

**FIGURE 6:**
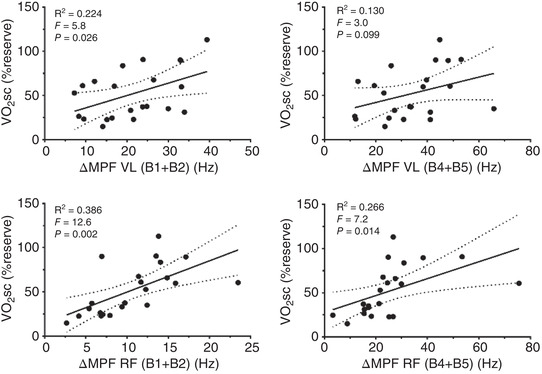
Relationship between ${\dot{V}}_{O_{2}}$ slow component (${\dot{V}}_{O_{2}\mathrm{sc}}$) and different mean power frequency (MPF) bands in the vastus lateralis (VL, upper panels; B1 + B2, *R* ^2^ = 0.224, *F* = 5.8, *P* = 0.026; B4 + B5, *R* ^2^ = 0.130, *F* = 3.0, *P* = 0.099) and rectus femoris (RF, lower panels; B1 + B2, *R* ^2^ = 0.386, *F* = 12.6, *P* = 0.002; B4 + B5, *R* ^2^ = 0.266, *F* = 7.2, *P* = 0.014) muscles when analysed separately. ∆MPF, difference between 12 min and zero. Left panels show lower bands (B1 + B2) and right panels show higher bands (B4 + B5); *n* = 11 participants.

A significant relationship between ${\dot{V}}_{O_{2}\mathrm{sc}}$ and RMS ∆ 12 min was found (sum of all muscles) (*R* ^2^ = 0.254, *F* = 6.8, *P* = 0.019). The ${\dot{V}}_{O_{2}\mathrm{sc}}$ presented a significant relationship with lower and higher MPF bands (∆ 12 min) analysing all muscles together (B1 + B2, *R* ^2^ = 0.382, *F* = 12.4, *P* = 0.002; B4 + B5, *R* ^2^ = 0.302, *F* = 8.6, *P* = 0.008; Figure 5) and when VL (B1 + B2, *R* ^2^ = 0.224, *F* = 5.8, *P* = 0.026; B4 + B5, *R* ^2^ = 0.130, *F* = 3.0, *P* = 0.099) and RF (B1 + B2, *R* ^2^ = 0.386, *F* = 12.6, *P* = 0.002; B4 + B5, *R* ^2^ = 0.266, *F* = 7.2, *P* = 0.014) muscles were analysed separately (Figure 6). There was no relationship between ${\dot{V}}_{O_{2}\mathrm{sc}}$ and MPF in the BF muscle (B1 + B2, *R* ^2^ = 0.126, *F* = 2.88, *P* = 0.105; B4 + B5, *R* ^2^ = 0.119, *F* = 2.69, *P* = 0.117). It is noteworthy that, the prediction of the variance in the ${\dot{V}}_{O_{2}\mathrm{sc}}$ was higher in the RF muscle than VL muscle in both MPF bands.

### 3.4 Muscle oxidative capacity ($m{\dot{V}}_{O_{2}}$) kinetics

The $m{\dot{V}}_{O_{2}}$ recovery rate constant (*K*) was 1.73 ± 0.4 min^−1^, with mean τ value of 36.3 ± 7.4 s. The variation coefficient for *K* was 12.6 ± 4.5% with an *R* ^2^ of 0.97 ± 0.02 for the adjustments. There was no relationship between τ of $m{\dot{V}}_{O_{2}}$ and ${\dot{V}}_{O_{2}\mathrm{sc}}$ for any intensity (HVY, *P* = 0.373; VHI, *P* = 0.904). A significant relationship was found between τ of $m{\dot{V}}_{O_{2}}$ and HHb_*A*_total_ (MOD, *r* = −0.533, *P* = 0.034; HVY, *r* = −0.637, *P* = 0.008; VHI, *r* = −0.531, *P* = 0.034) and HHb_end_ (VHI, *r* = −0.547, *P* = 0.028). Further, τ of $m{\dot{V}}_{O_{2}}$ was correlated with HHb_τ (*r* = 0.510; *P* = 0.044) and MRT (*r* = 0.620; *P* = 0.010) during HVY exercise. This means that a faster τ of $m{\dot{V}}_{O_{2}}$ could drive a faster HHb kinetics during exercise. Furthermore, when linear stepwise regression was performed to obtain the power of prediction from EMG activity variables, HHb MRT and pulmonary ${\dot{V}}_{O_{2}}$ τ, the τ of $m{\dot{V}}_{O_{2}}$ alone explained ∼50% of the variance in ∆torque 12 min for the VHI condition (*R* ^2^ = 0.477, *F* = 8.21, *P* = 0.019).

## 4 DISCUSSION

The main purpose of this study was to explain ${\dot{V}}_{O_{2}\mathrm{sc}}$ and muscle fatigue during cycling in different exercise intensity domains. Secondly, it aimed to verify the physiological mechanisms associated with the ${\dot{V}}_{O_{2}\mathrm{sc}}$ phenomenon. It was hypothesised that muscle fatigue would be related to muscle oxidative capacity, HHb kinetics and EMG activity. Secondly, it was hypothesised that muscle fatigue per se cannot explain the ${\dot{V}}_{O_{2}\mathrm{sc}}$ phenomenon.

The main outcome of this study was the demonstration, for the first time, that the additional O_2_ cost (i.e., ${\dot{V}}_{O_{2}\mathrm{sc}}$) and muscle fatigue (torque decreases) are associated with muscle activation heterogeneity. Furthermore, ${\dot{V}}_{O_{2}\mathrm{sc}}$ was temporally dissociated from muscle fatigue. Considering that muscle fatigue happened without significant decreases in EMG activity (RMS and MPF), it is possible to argue that ${\dot{V}}_{O_{2}\mathrm{sc}}$ seems not to be related to peripheral muscle fatigue either. Thus, the hypotheses of this study were accepted showing that muscle oxidative capacity and muscle activation heterogeneity partially explained the muscle fatigue, and further, the muscle fatigue per se is not a factor in explaining the ${\dot{V}}_{O_{2}\mathrm{sc}}$.

### 4.1 The reliability of torque measure with isokinetic efforts

No differences were observed for either peak torque (intraclass correlation coefficient = 0.99; typical error = 3.7%) or peak power output (intraclass correlation coefficient = 0.99; typical error = 4.2%) obtained during MIE in the pre‐exercise measurements (do Nascimento Salvador et al., 2018). In the present study the reliability of the torque assessments with isokinetic efforts (i.e., MIE) was confirmed. There were no differences within and between MIE performed during the baseline period or after the MOD condition in the different visits. This investigation showed no learning improvement or training effects on the MIE between different visits within conditions. It is important to note that neither RMS (indicating muscle activation) nor MPF (indicating motor unit (MU) recruitment) was influenced by condition or time during the MIE. These results could indicate that torque decreases observed mainly during VHI exercise were dissociated from a decrease in muscle activation and/or muscle fibre conduction velocity suggesting that decreases observed in muscle force production were originated probably after the neuromuscular junction or inside the muscle cell (corresponding to peripheric muscle fatigue).

### 4.2 Relation of torque with $m{\dot{V}}_{O_{2}}$ and HHb kinetics

It is noteworthy that during HVY exercise there was a small decrease in muscle force production (i.e., ∆torque 2 min), which was recovered at the end of exercise. Besides, during VHI exercise torque production decreases in the first 2 min but was stable after that until the end of exercise. The muscle fatigue analysed during the period of the slow component (i.e., ∆torque SC) in the HVY condition was related to the time constant of HHb kinetics (HHb_τ; *r* = 0.63; *P* = 0.011) and τ in the ${\dot{V}}_{O_{2}}$pulmonary kinetics (*r* = 0.54; *P* = 0.040) suggesting that the muscle fatigue in this condition could be partially explained by the velocity of oxidative pathways. These findings are in agreement with what was first demonstrated by Poole and colleagues by direct measurement (Poole et al., 1991). The ∆torque 2 min for the VHI condition was also related to HHb_end_ (*r* = −0.52; *P* = 0.046) indicating that a higher muscle fatigue leads to a lower HHb amplitude at the end of exercise. The TSI (the difference between final values and baseline values, 14.3 ± 4.0%) during VHI exercise was related to ∆torque 12 min (*r* = −0.53; *P* = 0.037), which points to an association between the O_2_ delivery and muscle fatigue. Likewise, the τ of $m{\dot{V}}_{O_{2}}$ explained ∼50% (*R* ^2^ = 0.47) of variance in ∆torque 12 min for the VHI condition. Despite that the micromatching of O_2_ delivery with O_2_ utilization heterogeneity cannot be assessed by the techniques used herein and may well be present, these results together show that muscle fatigue is partially due to phosphorylation oxidative capacity to produce ATP to sustain the activity.

Considering that the HHb_*A*_total_ was higher for VHI exercise leading to higher HHb_end_ values compared to HVY, and TSI was similar between conditions indicating that O_2_ delivery was not compromised during VHI, then a metabolic inefficiency inside the muscle cell is possibly linked to an oxidative energy production disturbance (Grassi, 2005; Korzeniewski & Rossiter, 2020; Rossiter et al., 2003). It is important to highlight that RPE was also related to ∆torque 12 min during HVY (14.4 ± 1.6 a.u.; *r* = 0.61; *P* = 0.016) and VHI exercise (17.8 ± 1.9 a.u.; *r* = 0.62; *P* = 0.014) showing that a ‘global fatigue’ is also a contributing factor to muscle fatigue.

### 4.3 Relationship between torque and muscle activation and recruitment

When analysed with all muscles together (i.e., VL, RF and BF) the recruitment of HTMUs explains ∼27% of variation in ∆torque 12 min independently of exercise intensity. However, there was no relation between ∆torque 12 min and LTMUs when all muscles together were analysed. This could indicate that the recruitment of less aerobic muscle fibres partially drives a greater muscle fatigue (Grassi et al., 2015). Interestingly, when just RF muscle was analysed, either LTMUs or HTMUs were related with ∆torque 12 min showing that for this specific musculature the recruitment of both muscle fibre types partially explains (∼30%) muscle fatigue. It is also important to state that ∆torque SC, was related to muscle fibre conduction velocity of LTMUs (*R* ^2^ = 0.23; *F* = 5.93; *P* = 0.024) and of HTMUs (*R* ^2^ = 0.35; *F* = 10.8; *P* = 0.004) during the same period, when all muscles were put together. The present study supports in part to the theory of random MU recruitment order (Dideriksen & Farina, 2013). Also, a recent paper (Okushima et al., 2020) has shown that the RF might present different deoxygenation profiles dependent upon specific facets of muscle architecture and patterns of diffusive and convective O_2_ transport as different muscle activation patterns. The present work also confirms the work of Cannon et al. (2021) that demonstrated that the greatest metabolic disturbance (i.e., [PCr]) was most commonly seen in the RF muscle during knee‐extensor exercise.

### 4.4 Lack of relationship between muscle fatigue and ${\dot{V}}_{O_{2}\mathrm{sc}}$

During mainly the past decade some authors have stated a relationship between muscle fatigue and ${\dot{V}}_{O_{2}\mathrm{sc}}$ (i.e., work inefficiency). The study of Cannon et al. (2011) apparently triggered a discussion about this, showing that high exercise intensities lead to a higher ${\dot{V}}_{O_{2}\mathrm{sc}}$ and increase the loss of peak power production (*R* ^2^ = 0.49). These authors also did not find differences in muscle torque production between minutes 3 and 8 during HVY or VHI exercise. The authors concluded that skeletal muscle fatigue precedes and is a requisite for the development of the ${\dot{V}}_{O_{2}\mathrm{sc}}$ despite the temporal dissociation between these two phenomena. The results from the present investigation confirm that even for VHI exercise there was no difference in muscle torque production between the 2nd and the 12th minute of cycling. The present results did not show significant decreases in torque production during HVY exercise after 2 min even with the observation of ${\dot{V}}_{O_{2}\mathrm{sc}}$. It is possible to argue that there was a small increase, but this leads to a higher O_2_ cost driving to a higher ${\dot{V}}_{O_{2}\mathrm{end}}$ than predicted during HVY exercise. Our results confirm the theory that if muscle fatigue influences the ${\dot{V}}_{O_{2}\mathrm{sc}}$ it precedes the phenomenon.

Recently, another important study on this topic (Keir et al., 2016) demonstrated a temporal relationship between peripheral muscle fatigue measured with electrostimulation (10/50 Hz ratio) in different visits of different durations and the magnitude of ${\dot{V}}_{O_{2}\mathrm{sc}}$ during cycling. It seems this was the first study to provide real evidence linking the higher O_2_ cost of exercise with changes in muscle fatigue. It was already stated that torque decreases in VHI exercise happen without changes in muscle activation (i.e., RMS) or muscle stimulation conduction velocity (i.e., MPF), possibly indicating a peripheral muscle fatigue in the present investigation. Contrary to Keir et al. (2016) the present work did not show a temporal association between muscle fatigue during VHI exercise and ${\dot{V}}_{O_{2}\mathrm{sc}}$. An important study on this topic was published more recently (Gajanand et al., 2020) replicating the experiments of Keir et al. (2016) but extending the duration of exercise. These authors found that the development of ${\dot{V}}_{O_{2}\mathrm{sc}}$ happened mainly between 2 and 10 min, during which neuromuscular properties were relatively stable, and peripheral muscle fatigue happened only after 20–30 min of exercise while ${\dot{V}}_{O_{2}\mathrm{sc}}$ was stable. The authors argued that the development of fatigue due to alterations of neuromuscular properties is not an essential requirement to elicit the ${\dot{V}}_{O_{2}\mathrm{sc}}$.

### 4.5 ${\dot{V}}_{O_{2}\mathrm{sc}}$ and lactate metabolism

The inconsistency between the findings mentioned above and the present results could be at least partially explained by the theory of Korzeniewski and Rossiter (2020). These authors stated that inorganic phosphate is the major contributor to the ${\dot{V}}_{O_{2}\mathrm{sc}}$ and, the additional ATP usage indeed starts when cytosolic inorganic phosphate exceeds a ‘critical’ concentration. Another study from the same authors (Korzeniewski & Rossiter, 2015) indicated that the mechanisms of each‐step activation of oxidative phosphorylation and inhibition of ATP supply by anaerobic glycolysis are associated with the ${\dot{V}}_{O_{2}\mathrm{sc}}$. Although with the present experimental approach it is not possible to check the factors linked to fatigue in the myocyte leading to high O_2_ cost during cycling, a positive relationship between ${\dot{V}}_{O_{2}\mathrm{sc}}$ and [La] was found. Despite this being the opposite of the findings of Poole et al. (1994), which did not support a role for lactate per se in driving the ${\dot{V}}_{O_{2}\mathrm{sc}}$ during intense exercise, these results partially confirm the theory stated above.

During the VHI cycling (probably near or above critical power) a steady state of [La] was not achieved leading to higher muscle and blood [La] values. Inside the muscle cell, inorganic phosphate concentration increases, and the pH is affected (Korzeniewski & Rossiter, 2020; Poole et al., 2016). This metabolic disturbance influences oxidative pathways and could be observed in the ${\dot{V}}_{O_{2}\mathrm{sc}}$. These findings are in accordance with previous studies stating that in the VHI exercise the ${\dot{V}}_{O_{2}\mathrm{sc}}$ may be explained by both a prolonged metabolic shifting between aerobic and glycolytic metabolism and a loss of efficiency over time (Colosio et al., 2020), and by decreased anaerobic energy contribution beyond 3 min during cycling exercise as demonstrated in the study of O'Connell et al. (2017).

### 4.6 Relation of ${\dot{V}}_{O_{2}\mathrm{sc}}$ with MPF in all muscles

When the EMG activity was analysed with all muscles together, the ${\dot{V}}_{O_{2}\mathrm{sc}}$ was partially explained by the muscle fibre conduction velocity of LTMUs and HTMUs independent of intensity (*R* ^2^ = 0.38 and *R* ^2^ = 0.30, respectively) showing evidence that any type of muscle fibres (i.e., type I and II) are recruited and result in a work inefficiency observed as a high cost of O_2_ during the activity. Interestingly, when just VL muscle was analysed just LTMUs were related to ${\dot{V}}_{O_{2}\mathrm{sc}}$ (*R* ^2^ = 0.22). However, when EMG activity of RF muscle was analysed, both LTMUs (*R* ^2^ = 0.39) and HTMUs (*R* ^2^ = 0.27) were associated to ${\dot{V}}_{O_{2}\mathrm{sc}}$ showing a higher power to explain the variation of this loss of work efficiency than with VL. Besides, MPF from the BF was dissociated from ${\dot{V}}_{O_{2}\mathrm{sc}}$. These results together indicate a muscle recruitment strategy during cycling exercise that results in the ${\dot{V}}_{O_{2}\mathrm{sc}}$. In other words, the ${\dot{V}}_{O_{2}\mathrm{sc}}$ seems to result from different sources inside muscle cells wherein, considering the abovementioned power to explain the ${\dot{V}}_{O_{2}\mathrm{sc}}$, the recruitment of type I fibres is also an important factor to be considered. This would provide a more outstanding contribution to the O_2_ high cost during cycling, and the VL and RF muscles play distinct roles.

The first paper to demonstrate directly evidence indicating that MU recruitment could explain the ${\dot{V}}_{O_{2}\mathrm{sc}}$ was Barstow et al. (1996). After that, many authors argued that the recruitment of less efficient muscle fibres leads to a higher O_2_ cost during exercise, although Barstow et al. (1996) found an inverse relationship between type I muscle fibres and ${\dot{V}}_{O_{2}\mathrm{sc}}$ using a muscle biopsy approach. Other authors using different methodologies also tried to confirm a relationship between less oxidative muscle fibres and ${\dot{V}}_{O_{2}\mathrm{sc}}$ (Borrani et al., 2001; Carter et al., 2004; Pringle et al., 2003). The work of Pringle et al. (2003) showed that the percentage of type I MUs was less when the ${\dot{V}}_{O_{2}\mathrm{sc}}$ was higher and vice versa during HVY and VHI exercise. Neither Barstow et al. (1996) nor Pringle et al. (2003) have shown an association between type II muscle fibres and ${\dot{V}}_{O_{2}\mathrm{sc}}$. Subsequent studies have confirmed that the dominant portion of ${\dot{V}}_{O_{2}\mathrm{sc}}$ has an origin in the MUs involved during exercise (Koga et al., 2005; Krustrup et al., 2008). The present paper shows that independent of exercise intensity, LTMUs are activated and explain almost 40% of this higher O_2_ cost variation during cycling, confirming the literature (Barstow et al., 1996; Pringle et al., 2003; Russell et al., 2002). The higher muscle fibre conduction velocity of LTMUs mainly from RF muscle is also associated with the decreases in torque production, which links a possible recruitment of type I muscle fibres with the ${\dot{V}}_{O_{2}\mathrm{sc}}$ and muscle fatigue during the exercise. Moreover, ${\dot{V}}_{O_{2}\mathrm{sc}}$ is not directly linked to muscle fatigue measured as loss of muscle force production but is indirectly linked to muscle fatigue considering all these factors discussed. These results are in agreement with the findings from Russell et al. (2002) who demonstrated that ${\dot{V}}_{O_{2}\mathrm{sc}}$ in trained subjects was best explained by citrate synthase activity (oxidative capacity of the contracting muscle) and in trained subjects combined with recreationally active subjects the ${\dot{V}}_{O_{2}\mathrm{sc}}$ correlated negatively with the percentage of type I muscle fibres.

In summary, this study confirmed that there is no temporal relationship between ${\dot{V}}_{O_{2}\mathrm{sc}}$ and the behaviour of muscle force production. This may be interpreted as indicating that muscle fatigue during heavy or very heavy cycling does not explain the ${\dot{V}}_{O_{2}\mathrm{sc}}$. However, the loss of torque production, the oxygenation changes in the ratio between $m{\dot{V}}_{O_{2}}$ and O_2_ delivery, a greater rating of perceived exertion, and a higher ${\dot{V}}_{O_{2}\mathrm{sc}}$, mainly during very heavy exercise, seem to share physiological mechanisms of similar origin. The work efficiency loss is bigger in the very heavy domain, and the changed anaerobic energy production is involved with the ${\dot{V}}_{O_{2}\mathrm{sc}}$ and muscle fatigue. The activation and recruitment of type I and II motor units mainly in the rectus femoris muscle could partially explain the additional O_2_ cost and muscle fatigue. The relationship of muscle fatigue and the ${\dot{V}}_{O_{2}\mathrm{sc}}$ with the muscle fibre conduction velocity of different muscles is dependent on muscle activation heterogeneity during cycling.

### 4.7 Limitations

Critical power is considered a ‘gold standard’ measure to define the boundary between heavy and very heavy domains (Poole et al., 2016). Critical power was not determined during this investigation, and it is one of the study's limitations. The 60% difference between GET and ${\dot{V}}_{O_{2}\mathrm{peak}}$ was used during this study avoiding the 50% value normally accepted as the upper boundary of the heavy domain for healthy subjects. It is impossible to affirm that all the participants were cycling in the exact zone above the critical power within the VHI domain, although it is necessary to consider that the mean values of VHI power output are really close to the RCP power output, indicating that at least the participants were cycling very near to the upper boundary of the heavy exercise domain. It is important to note that there is evidence in the literature positioning RCP in a higher power output than critical power during cycling (Leo et al., 2017).

This study did not use muscle biopsy as an approach to determine the motor units involved during the exercise. Instead, the EMG activity was analysed during cycling. The methodology used to observe the muscle activation (i.e., root‐mean‐squared) is widely accepted. Otherwise, the method to determine mean power frequency and the assumption that it could indicate the recruitment of different muscle fibre types is criticized elsewhere (Farina et al., 2002). Farina et al. (2002) argued that there are biophysical details that mask the possible association between EMG spectral variables and estimated conduction velocity. On the other side, von Tscharner and Nigg (2008) have affirmed that changes in spectral power frequency of muscle activities are task‐dependent, and are associated with changes in the selected muscle fibre types.

Notwithstanding, it is possible to find in the literature a group of authors from different places who have been using this methodological approach to investigate the muscle recruitment (Alonso et al., 2020; Borrani et al., 2001; Okushima et al., 2020; Wakeling et al., 2002). The specific evidence and arguments in defence of this method were published some time ago (Wakeling et al., 2002). During the EMG analysis of this study, arduous research in theoretical and practical terms was conducted to apply and reproduce the mean power frequency approach in a scientifically correct way. The purpose was to investigate in inferential mode the different muscle recruitment strategies during exercise.

## AUTHOR CONTRIBUTIONS

Conception and design of the work: Paulo Cesar do Nascimento Salvador, Diego Antunes, Eduardo Marcel Fernandes Nascimento, Luiz Guilherme Antonacci Guglielmo and Benedito Sérgio Denadai. Acquisition, analysis and interpretation of data for the work: Paulo Cesar do Nascimento Salvador, Diego Antunes, Eduardo Marcel Fernandes Nascimento, Luiz Guilherme Antonacci Guglielmo and Benedito Sérgio Denadai. Drafting the work or revising it critically for important intellectual content: Paulo Cesar do Nascimento Salvador and Benedito Sérgio Denadai. All authors have read and approved the final version of this manuscript and agree to be accountable for all aspects of the work in ensuring that questions related to the accuracy or integrity of any part of the work are appropriately investigated and resolved. All persons designated as authors qualify for authorship, and all those who qualify for authorship are listed.

## CONFLICT OF INTEREST

None.

## Supporting information

Statistical Summary Document

## Data Availability

The data that support the findings of this study are available from the corresponding author upon reasonable request.
