# Supplementary material for: Energy metabolism and muscle activation heterogeneity explain V˙O2 slow component and muscle fatigue of cycling at different intensities
Source: Exp Physiol. 2023 Jan 17;108(3):503–17. doi: 10.1113/EP090444 (PMC10103881; doi:10.1113/EP090444)
Supplement: Supplementary file 1 — Statistical Summary Document [file EPH-108-503-s001.pdf]

Experimental Physiology

Statistical Summary Document

Manuscript Title: **Energy metabolism and muscle activation heterogeneity explain Vo2 slow component and muscle fatigue of cycling at different intensities**

Authors: Paulo Cesar do Nascimento Salvador, Eduardo Marcel Fernandes Nascimento, Diego Antunes, Luiz Guilherme Antonacci Guglielmo, Benedito Sérgio Denadai.

Animal model used, if applicable:

Underlying hypothesis: It was hypothesised that 1) muscle fatigue would be related to muscle oxidative capacity, muscle fractional O_2 extraction and EMG activity; 2) $\dot{V}O_2$sc would be associated with muscle activation and recruitment and 3) muscle fatigue per se cc

Definitions of 'n': Number of participants

Statistical summary table:

Experimental question number	Finding/ conclusion	Experimental location/variable	Mean value	Standard Deviation	n	Exact P value	Figure/table in which data are presented	Units	Data comparisons	Statistical test	Any other experimental factors	Comments
Which physiological factors explain muscle fatigue?	Muscle fatigue in partially explained by muscle oxidative activity	Torque, power output, HHb kinetics and VO2m	VHI Δtorque 12 min 30.0 Nm; VO2m τ values of 36.3 s; VHI HHb_MRT 25.3 s; VHI HHb_END 90.2%	24.9 Nm; VO2m τ values 7.4 s;VHI HHb_MRT 6.7 s; VHI HHb_END 16.1%	16	0.019	Table 2 and 3	Nm, %PN and s	τV̇O2m vs. Δtorque 12min at VHI condition (R2 = 0.477, F = 8.21, p=0.019).	linear stepwise regression	N/A	We have summarised the main analysis and results here. It was performed many other analyses which are detailed in the text.
	Muscle fatigue is partially explained by muscle activation heterogeneity	Torque, power output, RMS and MPF from RF, VL and BF muscles	VL RMS at VHI 12 min 243.45 Hz; RF MPF (B4+B5) at VHI 33.81 Hz Δ12 min;	VL RMS at VHI 12 min 102.15 Hz; RF MPF (B4+B5) at VHI 16.82 Hz Δ12 min;	11	0.008	Table 3, figure 4 and 5	Nm, Hz	Δtorque 12min at VHI vs. higher MPF bands in RF (B4 + B5 = R2= 0.31; F = 8.82; p= 0.008)	Linear regression	N/A	We have summarised the main analysis and results here. It was performed many other analyses which are detailed in the text.
Measurements of EMG activity during cycling are associated with VO2sc?	Muscle activation (RMS) and muscle fibre conduction velocity are related with VO2sc	VO2sc, MPF from RF, VL and BF muscles	VO2sc 0.35; Δ12 min for B4+B5 at RF MPF at VHI 33.81 Hz; VL MPF at VHI 40.04 Hz; BF MPF at VHI 19.59 Hz;	VO2sc 0.14; Δ12 min for B4+B5 at RF MPF at VHI 16.82 Hz; VL MPF at VHI 15.37 Hz; BF MPF at VHI 7.91 Hz;	11	0.009	Table 1, figures 5 and 6	L.min-1 and Hz	Δtorque 12min at VHI vs. lowerMPF bands in RF muscle (B1 + B2 = R2 = 0.30; F = 8.41; p=0.009)	Linear regression	ANOVA two-way and Student t-test when applicable	We have summarised the main analysis and results here. It was performed many other analyses which are detailed in the text.
Torque decreases in an isokinetic maximal cycling effort (i.e muscle fatigue) are related to VO2sc phenomena?	There is no relationship between V̇O2sc and the behaviour of muscle force production.	Torque, power output and VO2sc	VHI Δtorque 12 min 30.0 Nm; VHI power output 12 min 1066.2 W; VO2sc 0.35;	VHI Δtorque 12 min 24.9 Nm; VHI power output 12 min 297.3 W; VO2sc 0.14;	16	0.588	Tables 1 and 3	Nm and L.min-1	Δtorque 12min at HVY and VHI vs. VO2sc	Linear regression	ANOVA two-way and Student t-test when applicable	We have summarised the main analysis and results here. It was performed many other analyses which are detailed in the text.
